# Supraglottic jet oxygenation and ventilation for obese patients under intravenous anesthesia during hysteroscopy: a randomized controlled clinical trial

**DOI:** 10.1186/s12871-019-0821-8

**Published:** 2019-08-14

**Authors:** Hansheng Liang, Yuantao Hou, Liang Sun, Qingyue Li, Huafeng Wei, Yi Feng

**Affiliations:** 1 Department of Anesthesiology, Peking University People’s Hospital, Beijing100044, Beijing, China; 2 Department of Anesthesiology and Critical Care, Hospital of the University of Pennsylvania, Philadelphia, PA 19104 USA

**Keywords:** Jet ventilation, Supraglottic, Obesity, Anesthesia, Gastric antrum, Ultrasound, Hysteroscopy

## Abstract

**Background:**

Supraglottic jet oxygenation and ventilation (SJOV) can effectively maintain adequate oxygenation in patients with respiratory depression, even in apnea patients. However, there have been no randomized controlled clinical trials of SJOV in obese patients. This study investigated the efficacy and safety of SJOV using WEI Nasal Jet tube (WNJ) for obese patients who underwent hysteroscopy under intravenous anesthesia without endotracheal intubation.

**Methods:**

A single-center, prospective, randomized controlled study was conducted. The obese patients receiving hysteroscopy under intravenous anesthesia were randomly divided into three groups: Control group maintaining oxygen supply via face masks (100% oxygen, flow at 6 L/min), the WNJ Oxygen Group with WNJ (100% oxygen, flow: 6 L/min) and the WNJ SJOV Group with SJOV via WNJ [Jet ventilator working parameters:100% oxygen supply, driving pressure (DP) 0.1 MPa, respiratory rate; (RR): 15 bpm, I/E; ratio 1:1.5]. SpO_2_, P_ET_CO_2_, BP, HR, ECG and BIS were continuously monitored during anesthesia. Two-Diameter Method was deployed to measure cross sectional area of the gastric antrum (CSA-GA) by ultrasound before and after SJOV in the WNJ SJOV Group. Episodes of SpO_2_ less than 95%, P_ET_CO_2_ less than 10 mmHg, depth of WNJ placement and measured CSA-GA before and after jet ventilation in the WNJ SJOV Group during the operation were recorded. The other adverse events were collected as well.

**Results:**

A total of 102 patients were enrolled, with two patients excluded. Demographic characteristics were similar among the three groups. Compared with the Control Group, the incidence of P_ET_CO_2_ < 10 mmHg, SpO_2_ < 95% in the WNJ SJOV group dropped from 36 to 9% (*P* = 0.009),from 33 to 6% (*P* = 0.006) respectively,and the application rate of jaw-lift decreased from 33 to 3% (*P* = 0.001), and the total percentage of adverse events decreased from 36 to 12% (*P* = 0.004). Compared with the WNJ Oxygen Group, the use of SJOV via WNJ significantly decreased episodes of SpO_2_ < 95% from 27 to 6% (*P* = 0.023), P_ET_CO_2_ < 10 mmHg from 33 to 9% (*P* = 0.017), respectively. Depth of WNJ placement was about 12.34 cm in WNJ SJOV Group. There was no significantly difference of CSA-GA before and after SJOV in the WNJ SJOV Group (*P* = 0.234). There were no obvious cases of nasal bleeding in all the three groups.

**Conclusions:**

SJOV can effectively and safely maintain adequate oxygenation in obese patients under intravenous anesthesia without intubation during hysteroscopy. This efficient oxygenation may be mainly attributed to supplies of high concentration oxygenation to the supraglottic area, and the high pressure jet pulse providing effective ventilation. Although the nasal airway tube supporting collapsed airway by WNJ also plays a role. SJOV doesn’t seem to increase gastric distension and the risk of aspiration. SJOV can improve the safety of surgery by reducing the incidence of the intraoperative involuntary limbs swing, hip twist and cough.

**Trial registration:**

Chinese Clinical Trial Registry. Registration number, ChiCTR1800017028, registered on July 9, 2018.

## Background

It is estimated that more than 100,000 patients receive hysteroscopy each year in China. Usually, hysteroscopy is accomplished under intravenous (IV) anesthesia or sedation without endotracheal intubation, primarily with IV propofol and remifentanil [1]. Endoscopic sedation and analgesia by propofol/remifentanil have been significantly increased during the past 10 years [2]. Remedial oxygenation by the jaw lift or pressurized mask ventilation is usually performed to manage anesthesia/sedation mediated respiratory depression, especially in obese patients. Supraglottic jet oxygenation and ventilation (SJOV) is aimed at oxygenation and ventilation in patient with depressed respiration or apnea, and have been demonstrated effective in difficult airway management without significant complications [3]. However, it is unclear whether SJOV can be used effectively and safely to maintain adequate oxygenation/ventilation in obese patients during hysteroscopy under intravenous sedation. The efficacy and safety of SJOV via nasopharyngeal approach in the obese patients needs further elucidated, although recent case reports [4, 5] and several clinical trials [6–8] delineated effectiveness of SJOV maintaining oxygenation in the non-obese patients. The Wei Nasal Jet Tube (WNJ, Well Lead Medical Co. Ltd., Guangzhou, China. number: 20170501) with an inner diameter of 5.0 mm, outer diameter of 7.5 mm, and a length of 18 cm is a newly invented nasal tube [9]. Here, we conducted a single-blind, prospective, randomized controlled study, and hypothesized SJOV using WNJ can reduce adverse events of hypoxia and hypoventilation in obese patients under IV anesthesia with propofol and remifentanil during hysteroscopy, without tracheal intubation.

## Methods

### Ethics, consent and permissions

This study was approved by the local Ethics Committee of Peking University People’s Hospital (No. 2018PHB036–01). Informed written consent was obtained from the obese patients who underwent hysteroscopy between July and September 2018. This study adhered to the Consolidated Standards of Reporting Trials (CONSORT) guidelines.

### Study design

**Fig. 1:**
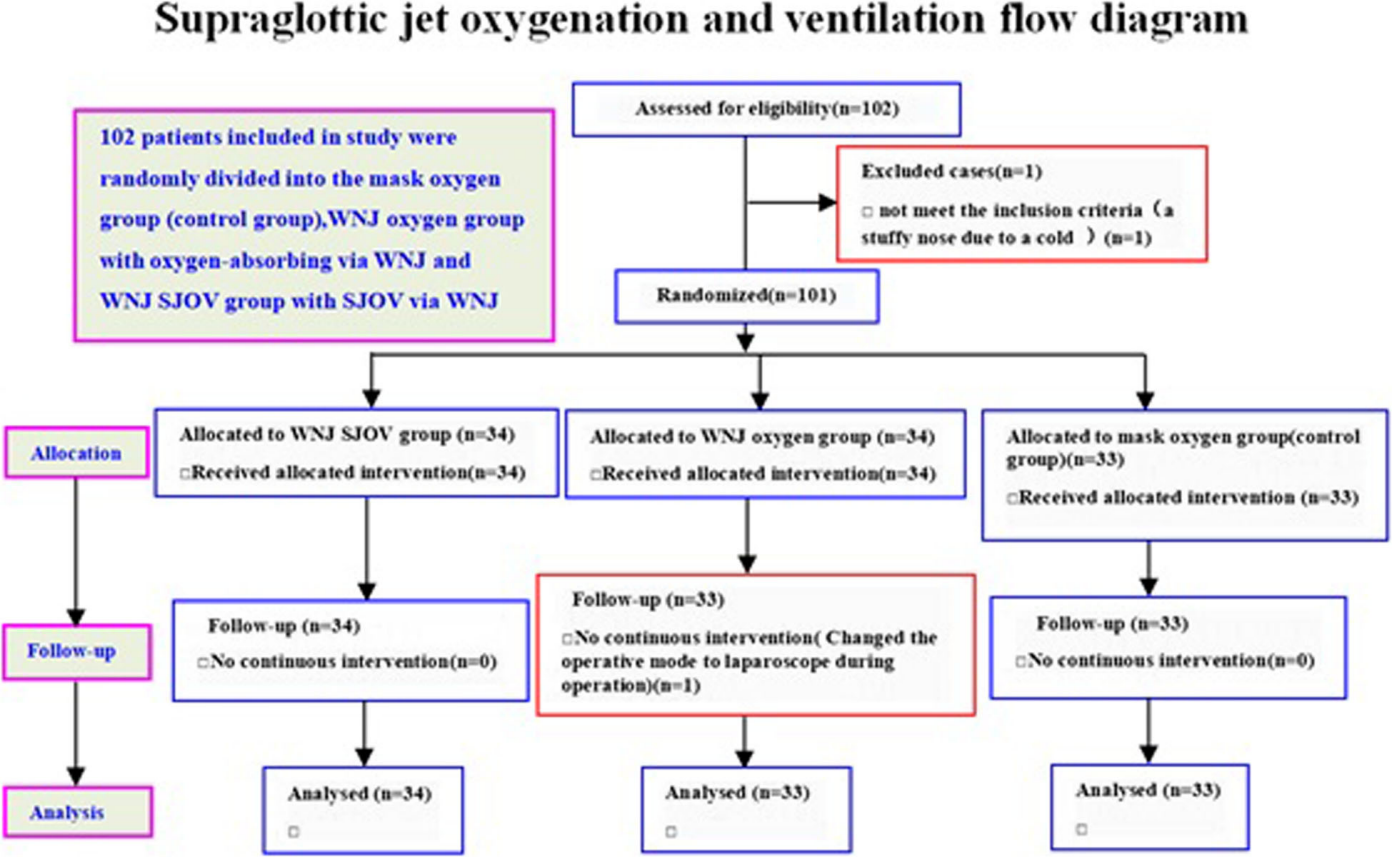
Supraglottic jet oxygenation and ventilation flow diagram. A total of 102 patients with BMI > 30 receiving hysteroscopy were randomly divided into three groups: the mask oxygen group (control group) maintaining oxygen absorption via face mask (*N* = 33), the WNJ oxygen group maintaining oxygen absorption via WNJ(*N* = 33) and the WNJ SJOV group maintaining SJOV via WNJ(*N* = 34). One patient in the control group was excluded because of stuffy nose due to a cold(*N* = 1). The other patient in WNJ oxygen group was abandoned intervention due to change of the operative mode during operation(*N* = 1)

Patients were randomized to the mask oxygen group (Control Group) maintaining oxygen absorption via face mask (100% oxygen, flow at 6 L/min), the WNJ oxygen group with WNJ (100% oxygen, flow at 6 L/min) and the WNJ SJOV group with SJOV via WNJ [Jet ventilator working parameters:100% oxygen supply, driving pressure (DP) 0.1 MPa, respiratory rate (RR): 15 bpm, I/E ratio 1:1.5]. The Sample Size was calculated by SAS, considering at least a 90 and 60% reduction in patients with SpO_2_ reduction in WNJ SJOV group and WNJ oxygen group compared to that in control group by the preliminary test. With a standard deviation of 0.8, and bilaterally equal to 0.05, or even 0.2 (power = 0.8), estimated value of each group should be 34 cases (*n* = 2(μ_α_ + μ_β_)^2^σ^2^/δ^2^ with 20% shedding rate). The randomization was performed by random number table from the SPSS23.0, and the blinding was completed by one medical student and two anesthesiologists. Patients were labeled with WNJ SJOV, WNJ or Control by One anesthesiologist, and the other anesthesiologist administered the intravenous anesthesia and maintained oxygenation. The flow diagram of this study was shown in Fig. 1.

### Patients

In-patients receiving routine hysteroscopy under IV anesthesia with propofol and remifentanil were recruited. Inclusion criteria were as follows: (1) 18 yrs. < age < 65 yrs.; (2) BMI > 30 kg/m^2^; (3) Fasting for 8 h and no water for 4 h before surgery; (1) ASA class: I-II classes. Exclusion criteria were as follows: (1) Epistaxis; (2) Nasal stenosis; (3) Long-term use of anticoagulants; (4) Rhinitis episodes; (5) Severe reflux disease; (6) History of severe respiratory, cardiovascular and cerebrovascular diseases.

### Anesthesia

**Fig. 2:**
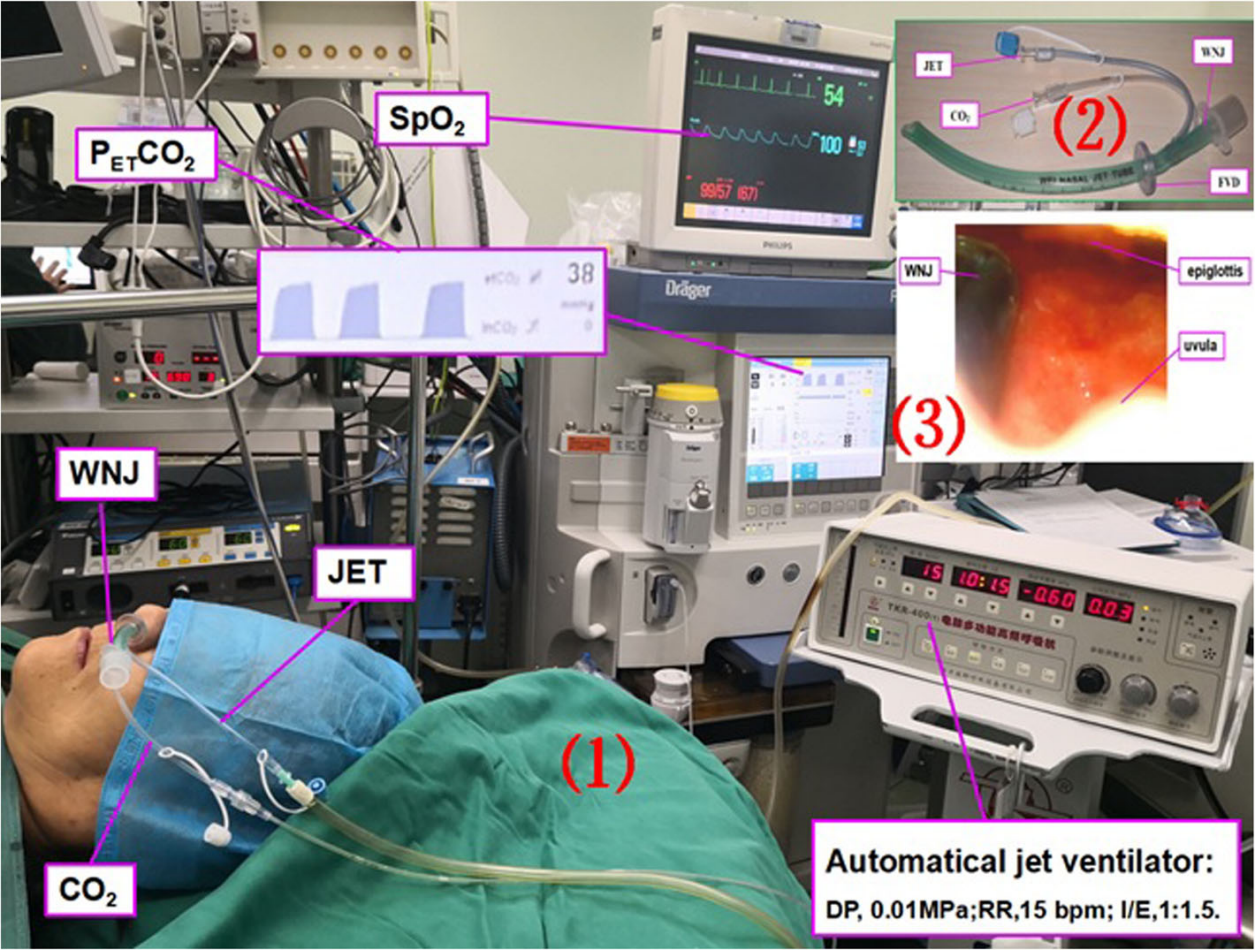
(1) The scene of supraglottic jet oxygenation and ventilation (SJOV) via WNJ with or without spontaneous breathing. SJOV could maintain oxygen saturation and carbon dioxide exhalation. P_ET_CO_2_ = End-tidal carbon dioxide partial pressure; SpO_2_ = pulse oxygen saturation;D*P* = driving pressure;RR = respiratory rate; I/E ratio = inhalation/exhalation ratio. (2) Wei Nasal Jet tube (WNJ), which has two channels built inside the tube wall for jet ventilation and the end-tidal pressure of CO_2_ monitoring, respectively. FVD = fixed valve of depth. (3) The position of the WNJ into the laryngopharynx observed by nasal fiberoptic scope. The depth of placement of the WNJ was about equivalent to the distance from the alar to the ipsilateral earlobe. The best site for WNJ insertion under fiber bronchoscope was between the epiglottis and uvula

Induction of anesthesia was conducted by IV injection of propofol (1.5–2 mg/kg) and remifentanil (0.5 μg/kg). Anesthesia was maintained by continuous IV infusion of propofol (3-5 mg/kg/h) and remifentanil (0.05–0.08μg/kg/ min). After anesthesia induction, the face mask in control group, the WNJ in WNJ oxygen group and WNJ SJOV group were placed appropriately straightway. Before putting WNJ into the unobstructed nostril of patients, a paraffin oil cotton swab was used to clear the nasal cavity and about 1 ml lidocaine ointment was smeared on the tip of WNJ (the depth was equivalent to the distance from the alar to the ipsilateral earlobe [3]. The jet catheter of the WNJ was connected to an automated jet ventilator-TKR-400 (Well Lead Medical Equipment Ltd. Guangzhou, China.) (Fig. 2). Bispectral index (BIS) was maintained at 45–60 [additional propofol (0.3–0.5 mg/kg) was given with one bolus infusion if needed] and SpO_2_ was maintained above 95%. A dose of ephedrine (3–6 mg) was administered as needed in order to maintain the mean arterial pressure above 55 mmHg. Remedial measures were executed immediately in the setting of oxygen saturation (SpO_2_) < 95%, including adjusting the WNJ position (1 cm deep or shallow) in the WNJ SJOV group and the WNJ oxygen group, and taking jaw-lift maneuver in the three groups. Mask pressurized ventilation was used to provide oxygen only when SpO_2_ < 90% happened in all the three groups.

### Intraoperative monitoring

**Fig. 3:**
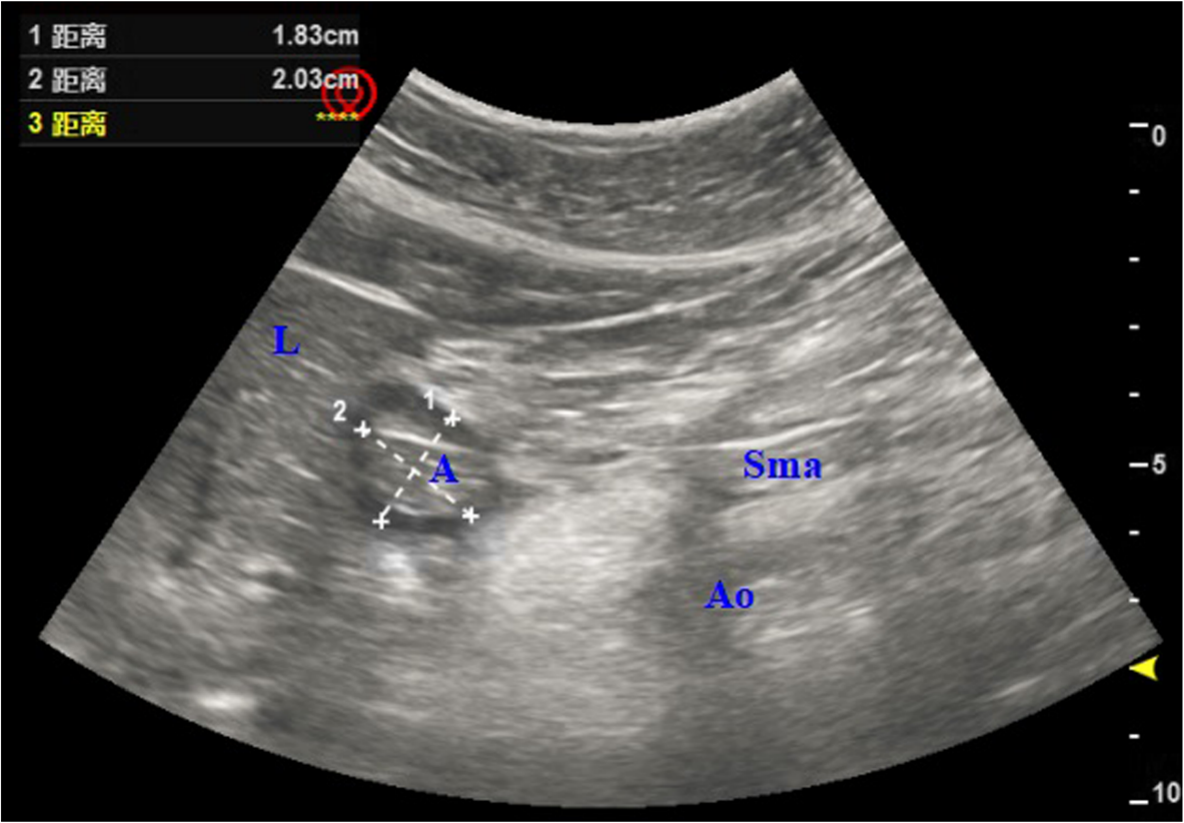
Two-Diameter Method was carried out to measure antral CSA by ultrasound before and after SJOV in the WNJ SJOV group. GA = Gastric antrum, Ao = aorta, Sma = superior mesenteric artery, L = liver. The antero-posterior (AP) antral diameter was expressed as dotted line 1,The cranio-caudal (CC) antral diameter was represented by dotted line 2,and π was 3.1416 during CSA = (AP × CC × π)/4

We continuously monitored following parameters during the anesthesia: pulse SpO_2_, End-tidal carbon dioxide partial pressure (P_ET_CO_2_), mean blood pressure (MBP), heart rate (HR), electrocardiogram (ECG) and BIS. SpO_2_ < 95% and P_ET_CO_2_ < 10 mmHg were considered hypoxic adverse event and hyperventilation, respectively [10]. Two-Diameter Method was used to measure antral cross-sectional area of gastric antrum (CSA-GA) by ultrasound [Vivid, GE MEDICAL SYSTEMS CO.,LTD, China] with patients in the supine position before and after SJOV in the WNJ SJOV group, using the sagittal plane measurement at the xiphoid process level, with the help of the anatomical markers (the superior mesenteric artery,the left liver lobe and abdominal aorta) (Fig. 3). The diameter of antero-posterior and cranio-caudal antral was expressed as AP and CC, respectively, with a π of 3.1416 during CSA = (AP × CC × π)/4. Stomach volume was estimated and calculated by estimated stomach volume (ESV) = 27.0 + 14.6 × CSA-1.28 × age (yrs) [11, 12].

Episodes of SpO_2_ less than 95% and P_ET_CO_2_ less than 10 mmHg during the operation were recorded. The other adverse events such as hip twist, cough, nasal bleeding and remedial interventions such as jaw-lift, pressure mask, depth of WNJ placement were also noted. Types and time of surgery, recovery time of anesthesia, dose of anesthetics, measured CSA-GA before and after jet ventilation in the WNJ SJOV group were also recorded.

### Statistical analysis

The measurement data was expressed as mean ± SD and the count data was presented as the number and percentage. Kruskal-wallis single factor ANOVA test was used to compare the difference of time of surgery, recovery time of anesthesia, dosage of anesthetics between different groups. The data about depth of WNJ placement between the WNJ SJOV and the WNJ oxygen groups was analyzed by Wilcoxon rank sum test. Independent sample t-test was used for comparing the CSA-GA and ESV before and after jet ventilation in the WNJ SJOV group. Categorical variables as the cases of SpO_2_ less than 95%, P_ET_CO_2_ less than 10 mmHg, hip twist, nasal bleeding, and patients requiring jaw-lift, pressured mask ventilation, as well as other perioperative adverse reactions were all analyzed withchi-square test and Fisher’s exact test. All data was analyzed with SPSS23.0 statistical software (SPSS Inc., Chicago, IL, USA). A value of *p* < 0.05 was considered the difference was statistically significant.

## Results

A total of 102 patients were enrolled. One patient in the control group was excluded because of stuffy nose due to catching a cold, and another patient in WNJ oxygen group was removed because of change of the operative mode during operation. All the patients tolerated the hysteroscopy well. There were no serious adverse events (i.e. aspiration, laryngospasm, nasal bleeding post operation, barotrauma and death).

### Clinical characteristics of study population

The clinical characteristics of patients was represented in Table 1. Age, height, weight, BMI, ASA classification, airway-related parameters, (including mouth opening degree, thyromental distance, neck circumference, Mallampati class), snore history, and obstructive sleep apnea hypoventilation syndrome (OSAHS) were compared. Data of the surgical and anesthesia procedure, adverse events and remedial interventions were analyzed in Table 2.

**Table 1:** General information of patients in the three groups and the surgical types. The differences among the three groups and between each two groups were not statistically significant(*p* > 0.05)

Characteristic (mean ± SD)	mask oxygen(I) (*N* = 33)	WNJ oxygen (II) (*N* = 33)	WNJ SJOV (III) (*N* = 34)
Age (yr.)	44.7 ± 11.65	44.1 ± 12.86	43.8 ± 15.22
Height (cm)	155.6 ± 5.56	154.4 ± 7.23	156.3 ± 8.23
Weight (kg)	80.3 ± 6.05	79.2 ± 10.34	80.5 ± 5.17
BMI (kg.m^−2^)	33.18 ± 2.87	33.23 ± 3.22	32.97 ± 2.36
mouth opening(I)(II)(III)(IV)	(32)(1)(0)(0)	(33)(0)(0)(0)	(33)(1)(0)(0)
thyromental Distance(I)(II)(III)	(27)(5)(1)	(28)(5)(0)	(30)(4)(0)
neck circumference(I)(II)(III)	(11)(19)(3)	(13)(18)(2)	(10)(20)(4)
mallampati class (I)(II)(III)(IV)	(8)(23)(2)(0)	(7)(22)(4)(0)	(8)(22)(4)(0)
Snore history [*n* (%)]	11 (33%)	13 (39%)	13 (38%)
OSAHS [*n* (%)]	0 (0%)	0 (0%)	1 (3%)
ASA(I)(II)	(28)(5)	(30)(3)	(30)(4)
surgical types of hysteroscopy			
TCRP [*n*(%)]	4 (12)	5 (15)	6 (18)
TCRM [*n*(%)]	6 (18)	5 (15)	6 (18)
TCRS [*n*(%)]	11 (33)	12 (36)	11 (32)
TCRA [*n*(%)]	12 (36)	11 (33)	11 (32)

*BMI* body mass index; Mouth opening(I/II/III/IV):I > 4.0 cm, II 2.5–3.0 cm, III 1.2–2.0 cm, and IV < 1.0 cm; Thyromental distance (I/II/III): I > 6.5 cm, II 6–6.5 cm, and III < 6 cm; Neck circumference(I/II/III): I < 35 cm, II 35–41 cm, and III > 41 cm; *OSAHS* obstructive sleep apnea hypoventilation syndrome, *SJOV* supraglottic jet oxygenation and ventilation, *WNJ* Wei nasal jet tube, *TCRP* transcervical polyp resection, *TCRM* transcervical hysteroscopy fibroid resection, *TCRS* transcervical resection of septa, *TCRA* transcervical resection of adhesions

**Table 2:** Data about the procedure, drugs dosage, adverse events and remedial interventions. Compared with the mask oxygen or WNJ oxygen groups, the use of SJOV via WNJ during the surgery significantly decreased the total percentage of adverse events and surgical time, cases of SpO_2_ < 95% and P_ET_CO_2_ < 10 mmHg, and the application rate of jaw-lift

Monitored variables [*n* (%)]	mask oxygen(I) (*N* = 33)	WNJ oxygen (II) (*N* = 33)	WNJ SJOV (III) (*N* = 34)	*P*-Value (I VS II)	*P*-Value (I VS III)	*P*-Value (II VS III)	*P*
Surgical time (min)	24.28 ± 10.18	23.19 ± 9.72	22.56 ± 5.91	0.053	0.013	0.053	0.027 (Kruskal-wallis)
Anesthesia recovery (min)	14.73 ± 5.59	13.22 ± 3.73	13.97 ± 4.12	0.068	0.217	0.866	0.061 (Kruskal-wallis)
WNJ placement depth (cm)	–	12.22 ± 0.54	12.34 ± 0.47	–	–	0.087^a^	–
Propofol dose (mg)	207.01 ± 62.85	212.57 ± 51.44	225.01 ± 48.63	1.000	0.002	< 0.001	< 0.001 (Kruskal-wallis)
Remifentanil dose (μg)	32.28 ± 6.18	33.02 ± 8.27	33.21 ± 4.97	0.059	0.244	0.196	0.079 (Kruskal-wallis)
Ephedrine dose (mg)	5.14 ± 1.03	5.23 ± 1.16	5.25 ± 1.10	0.516	0.417	0.975	0.022 (Kruskal-wallis)
Total adverse events	12 (36)	13 (39)	4 (12)	1.000	0.004	0.002	0.013
Intra-operation							
SpO_2_ < 95%	11 (33)	9 (27)	2 (6)	0.789	0.006	0.023	0.017
P_ET_CO_2_ < 10 mmHg	12 (36)	11 (33)	3 (9)	1.000	0.009	0.017	0.019
Jaw-lift	11 (33)	10 (30)	1 (3)	1.000	0.001	0.003	0.004
Mask pressurized ventilation	5 (15)	3 (9)	0 (0)	0.708	0.025	0.114	0.071
Oropharyngeal tube	2 (6)	0 (0)	0 (0)	0.492	0.239	–	0.126
Nasal bleeding	0 (0)	1 (3)	2 (6)	1.000	0.493	1.000	0.369
Cough	3 (9)	1 (3)	1 (3)	0.613	0.356	1.000	0.420
Laryngospasm	0 (0)	0 (0)	0 (0)	–	–	–	–
Aspiration	0 (0)	0 (0)	0 (0)	–	–	–	–
Hip twist	2 (6)	1 (3)	0 (0)	1.000	0.239	0.493	0.348
Bradycardia	9 (27)	8 (24)	4 (12)	1.000	0.132	0.217	0.254
Tachycardia	2 (6)	1 (3)	0 (0)	1.000	0.239	0.493	0.348
Hypertension	5 (15)	4 (12)	1 (3)	1.000	0.105	0.197	0.221
Hypotension	1 (3)	1 (3)	3 (9)	1.000	0.614	0.614	0.453
Post-operation							
Nausea or Vomiting	1 (3)	2 (6)	1 (3)	1.000	1.000	0.614	0.761
Pharyngalgia	2 (6)	3 (9)	3 (9)	1.000	1.000	1.000	0.881
Xerostomia	2 (6)	3 (9)	4 (12)	1.000	0.427	0.709	0.488
Nasal bleeding	0 (0)	0 (0)	0 (0)	–	–	–	–
Barotrauma	0 (0)	0 (0)	0 (0)	–	–	–	–

SpO_2_: pulse oxygen saturation; P_ET_CO_2_: End-tidal carbon dioxide partial pressure; SJOV:supraglottic jet oxygenation and ventilation; WNJ: Wei nasal jet tube

### Primary outcome

#### Adverse events of hypoxia and hypoventilation

The majority of patients had shown good wave form and good values of P_ET_CO_2_ during SJOV. Compared with the Control Group, the incidence of P_ET_CO_2_ < 10 mmHg,SpO_2_ < 95% in the WNJ SJOVgroup dropped from 36 to 9% (*P* = 0.009), from 33 to 6% (*P* = 0.006) respectively, and the application rate of jaw-lift decreased from 33 to 3% (*P* = 0.001), and the total percentage of adverse events decreased from 36 to 12% (*P* = 0.004). Compared with the WNJ Oxygen Group, the use of SJOV via WNJ significantly decreased episodes of SpO_2_ < 95% from 27 to 6% (*P* = 0.023), P_ET_CO_2_ < 10 mmHg from 33 to 9% (*P* = 0.017), respectively. There were no significant differences in episodes of SpO_2_ < 95% and P_ET_CO_2_ < 10 mmHg between the WNJ Oxygen Group and the Control Group (*P* = 0.789 and *P* = 1.000). (Table 2).

## Secondary outcome

### Incidence of the nasal bleeding, cough and hip twist

There were three cases of nasal bleeding (a small amount of blood attached WNJ) during operation in this study. One occurred in WNJ oxygen group, the other two cases happened in WNJ SJOV group. On the second day after surgery, these three patients had no other symptoms except feeling slight dry and itching in the throat.

There were no significant differences in the cough and hip twist among three groups (*P* = 0.420 and *P* = 0.348). However, compared with the Control Group, SJOV could slightly decrease the incidence of cough from 9 to 3% (*P* = 0.356) and hip twist from 6 to 0% (*P* = 0.239) (Table 2).

### Incidence of the nausea or vomiting, pharyngalgia, and xerostomia after surgery, changes of CSA-GA and ESV

There were no significant differences in the nausea or vomiting, pharyngalgia and xerostomia among three groups after surgery (*P* = 0.761, *P* = 0.488 and *P* = 0.881).(Table 2). The changes of the CSA-GA and ESV were not significant before and after supraglottic jet ventilation in the WNJ SJOV Group (*P* = 0.234 and *P* = 0.777). (Table 3).

**Table 3:** Two-Diameter Method was used to measure CSA-GA by ultrasound. Stomach volume was estimated and calculated by ESV = 27.0 + 14.6 × CSA-1.28 × age (years). Compared with before jet ventilation, CSA-GA and ESV after jet ventilation had not been increased in the WNJ SJOV group (*p* > 0.05)

Monitored variables	Before WNJ SJOV (*N* = 34)	After WNJ SJOV (*N* = 34)	P-Value (before VS after)
CSA-GA (cm^2^)	3.32 ± 0.59	3.34 ± 0.56	0.234
ESV (ml)	18.78 ± 6.68	18.89 ± 6.59	0.777

*CSA-GA* cross sectional area of the gastric antrum, *ESV* Estimated stomach volume, *SJOV* supraglottic jet oxygenation and ventilation, *WNJ* Wei nasal jet tube

### Propofol dosage

Compared with the Control Group and the WNJ Oxygen Group, respectively, the dosage of propofol in the WNJ SJOV Group increased significantly (*P* = 0.002, *P* < 0.001).

## Discussion

Hysteroscopy is one of the most common procedures in gynecology [1]. Involuntary limbs swing and hip twist should be avoided during hysteroscopy surgery [13], considering that it not only increases potential risk of uterine perforation but adding extra burden to operation. Therefore, sufficient depth of anesthesia and analgesic intensity is required. Admittedly, intraspinal anesthesia can provide adequate analgesia and satisfactory patient cooperation, but it is not conducive to rapid postoperative recovery of patients [14]. With the application of propofol and remifentanil in clinical practice, short IV anesthesia is possible and can be easily accepted by surgeons and patients. At present, IV anesthesia becomes a trend in hysteroscopy [1]. Majholm, an obstetrician and gynecologist in Denmark, believed that although the time of hysteroscopy surgery under non-intubation IV anesthesia was similar to that under intraspinal anesthesia or intubation general anesthesia, the length of hospital stay reduced and patients’ satisfactions were improved [15]. However, while providing satisfactory anesthesia and rapid patients’ recovery, anesthesiologists must guarantee a difficult task, that is adequate oxygenation and ventilation of patients. Therefore, the key to anesthesia management in hysteroscopy surgery is how to balance the short time, analgesic intensity and anesthesia depth with airway safety of patients. Shallow anesthesia is unable to meet surgery needs, however, deep anesthesia may lead to significant airway collapse. When the depth of anesthesia is sufficient for the operation, supplemental oxygen was proved to be an effective intervention measure to improve oxygenation and ventilation. These common measures include nasal catheter, mask, jaw-lift, or pressurized mask ventilation, which require the patient to maintain an autonomous breathing. Usually, pressurized mask ventilation is used as the last attempt in the settings of non-artificial airway assisted-ventilation [2]. However, this ventilation mode is easy to make stomach flatulence, and increase the risk of gastric reflux and aspiration [16]. Obesity is a multisystem, chronic, proinflammatory disorder, and specific care is needed for airway management [17]. Leakage is likely to occur when airway pressure is more than 20cmH_2_O for the patients with obesity under intravenous general anesthesia using laryngeal mask, and ventilation and oxygenation are affected. When the airway pressure exceeds 25cmH_2_O, it is easy to increase gastric distension and the risk of aspiration [18].

Transnasal humidified rapid insufflation ventilatory exchange (THRIVE) is a new approach to enhance oxygenation [19]. THRIVE has the advantage of increasing oxygen concentration, removing carbon dioxide from the ineffective chamber, and maintaining positive airway pressure, thus improving lung compliance and reducing upper airway obstruction [20]. However, in terms of upper airway obstruction, jet ventilation, which was developed in 1967, has the same enhanced oxygenation advantage as THRIVE, although they work in different ways [21, 22].

Jostrand from Sweden first introduced the technology of using 60 to 100 breaths per minute, which was called high frequency positive pressure ventilation (HFPPV) [23]. Klein studied the HFPPV and renamed the system as high frequency jet ventilation (HFJV) [24]. Since then, HFJV has become a technique to maintain ventilation. The application of this technique enables rapid pulsation gas to enter the respiratory tract through a narrow jet tube under low pressure [25, 26]. HFJV has three characteristics [27–29], open system, high-frequency (> 60 bpm) and low tidal volume. Transtracheal jet ventilation (TTJV) is one of the popular methods of emergency airway management in the ASA guide, but barotrauma is a severe complication that damper the excitement to use it [30–32]. The characteristics of supraglottic jet ventilation (SJV) are that can be used as the ventilation of emergency airway, and auxiliary oxygenation of difficult airway, with more open ventilating system, less complications, and low requirement for spontaneous breathing [33]. Advantages of WNJ are jetting ventilation via side hole, releasing gas from the main hole and surrounding upper airway space, and reduction of barotrauma.

In addition to observing chest fluctuation, P_ET_CO_2_ can also be monitored [34] [Fig. 2(2)]. In this study, we successfully applied SJOV via WNJ to decrease the episodes of hypoxia and hypoventilation for the patients receiving hysteroscopy surgery under IV anesthesia by propofol and remifentanil. This efficient oxygenation may be accomplished primarily by jet ventilation, though nasal airway tube supporting collapsed airway also plays a role. Additionally, SJOV was seemed to improve the safety of surgery by reducing the incidence of the intraoperative involuntary limbs swing and hip twist, cough, and stomach flatulence.

The scene of SJOV via WNJ was shown in Fig. 2(1). With or without spontaneous breathing, both oxygenation and carbon dioxide exhalation were well maintained. The depth of placing the WNJ was equivalent to the distance from the alar to the ipsilateral earlobe. Meanwhile, we located the front-end of WNJ by fibro bronchoscope and found that the best site for WNJ oxygenation was between epiglottis and uvula in the WNJ SJOV Group. The distance was about 12.34 cm [Fig. 2(3)]. Oxygenation effect can be maintained better in the WNJ SJOV Group than that in the WNJ Oxygen Group, although the same WNJs were used for the patients in both groups. Accordingly, we believe the role of maintaining oxygenation was mainly jet ventilation rather than propping up the collapsed airway with or without spontaneous respiration. For obese patients, it was usually difficult to perform hysteroscopy surgery under general IV anesthesia without intubation and maintain proper intensity of spontaneous breathing without respiratory depression under the guarantee of surgical safety. Therefore, WNJ SJOV had more advantages in enhancing oxygenation than WNJ alone.

Theoretically, SJV can make the WNJ front end swing, which may cause damage to the throat soft tissue. However, we did not find such kind of swing under the fiberoptic bronchoscopy. The possible reasons were that the curved part of the nasal cavity of WNJ weakened the airflow impact, and the jet ventilation through the WEJ side hole reduced the airflow and the airflow pressure. SJV via WNJ (WNJ SJV) could provide adequate ventilation, although it is less effective than transtracheal jet ventilation (TTJV). However, WNJ SJV could significantly reduce the complications of TTJV, such as barotrauma. WNJ SJV within 25 min did not increase incidence of postoperative laryngopharyngeal pain, cough and ability of discharge of sputum, suggesting that this technique may not produce inflammatory reaction caused by damage of airway mucosa and throat soft tissue.

Although the mechanisms about the respiratory depressant effect of propofol have not been fully explained, it is clear that propofol causes the respiratory depressant effect in a dose-dependent manner [35]. When mask/nasopharyngeal tube oxygen is used to maintain oxygenation under propofol sedation, propofol dosage is often reduced involuntary due to respiratory depressant, which will increase the incidence of the intraoperative involuntary limbs swing, hip twist and cough. The respiratory depressant effect may be not worried with/without spontaneous respiration during SJOV via WNJ to maintain oxygenation under propofol sedation. The short duration of hysteroscopy and the small total dosage of propofol which did not lead to the difference in anesthesia recovery, although there were differences in the dosage of propofol among the three groups.

Although it had been reported that SJV can maintain 1 h oxygenation, but it was not suitable for longer time application [8]. Appropriate use time and whether it will cause airway mucosal inflammation remains to be observed in large-sample multi-center randomized controlled trials in the future. Previous anesthesiologists criticize that this methods may not be sensitive enough to monitor gastric volume and extension by ultrasound. The CSA-GA in the evaluation of gastric flatulence may seemed to have poor quantitative accuracy, but it could provide some qualitative reference value in clinical application, so making efforts to explore this field was required in the future work.

The shortcomings of this trial are reflected in the small sample size, which cannot fully reflect the real situation of adverse reactions. The position of the patient during ultrasound examination is not the optimal position.

## Conclusions

SJOV can effectively and safely maintain adequate oxygenation in obese patients under intravenous anesthesia without intubation during hysteroscopy. This efficient oxygenation may be mainly attributed to supplies of high concentration oxygen to the supraglottic area, and the high pressure jet pulse providing effective ventilation. Although the nasal airway tube supporting collapsed airway by WNJ also plays a role. SJOV doesn’t seem to increase gastric distension and the risk of aspiration. SJOV can improve the safety of surgery by reducing the incidence of the intraoperative involuntary limbs swing, hip twist and cough.

## Data Availability

The datasets used and/or analyzed during the current study are available from the corresponding author on reasonable request.

## Abbreviations

Ao: aorta
AP: Antero-posterior
ASA: American Society of Anesthesiologists
BIS: Bispectral index
BMI: body mass index
CC: cranio-caudal
CONSORT: Consolidated standards of reporting trials
CSA-GA: Cross sectional area of the gastric antrum
DP: Driving pressure
ECG: Electrocardiogram
ESV: Estimated stomach volume
FVD: Fixed valve of depth
GA: Gastric antrum
HFJV: High frequency jet ventilation
HR: Heart rate
I/E ratio: Inhalation/exhalation ratio
IV: Intravenous
L: Liver
MBP: Mean blood pressure
OSAHS: Obstructive sleep apnea hypoventilation syndrome
P_ET_CO_2_: End-tidal carbon dioxide partial pressure
RR: Respiratory rate
SJOV: Supraglottic jet oxygenation and ventilation
SJV: Supraglottic jet ventilation
Sma: Superior mesenteric artery
SpO_2_: Pulse oxygen saturation
TCRA: Transcervical resection of adhesions
TCRM: Transcervical hysteroscopy fibroid resection
TCRP: Transcervical polyp resection
TCRS: Transcervical resection of septa
THRIVE: Transnasal humidified rapid insufflation ventilatory exchange
TTJV: Transtracheal jet ventilation
WNJ: Wei Nasal Jet tube
